# Protein domain organisation: adding order

**DOI:** 10.1186/1471-2105-10-39

**Published:** 2009-01-29

**Authors:** Sarah K Kummerfeld, Sarah A Teichmann

**Affiliations:** 1 Department of Developmental Biology, 279 Campus Dr, Stanford, 94305, CA, USA; 2 MRC Laboratory of Molecular Biology, Hills Rd, Cambridge, CB2 2QH, UK

## Abstract

**Background:**

Domains are the building blocks of proteins. During evolution, they have been duplicated, fused and recombined, to produce proteins with novel structures and functions. Structural and genome-scale studies have shown that pairs or groups of domains observed together in a protein are almost always found in only one N to C terminal order and are the result of a single recombination event that has been propagated by duplication of the multi-domain unit.

Previous studies of domain organisation have used graph theory to represent the co-occurrence of domains within proteins. We build on this approach by adding directionality to the graphs and connecting nodes based on their relative order in the protein. Most of the time, the linear order of domains is conserved. However, using the directed graph representation we have identified non-linear features of domain organization that are over-represented in genomes. Recognising these patterns and unravelling how they have arisen may allow us to understand the functional relationships between domains and understand how the protein repertoire has evolved.

**Results:**

We identify groups of domains that are not linearly conserved, but instead have been shuffled during evolution so that they occur in multiple different orders. We consider 192 genomes across all three kingdoms of life and use domain and protein annotation to understand their functional significance.

To identify these features and assess their statistical significance, we represent the linear order of domains in proteins as a directed graph and apply graph theoretical methods. We describe two higher-order patterns of domain organisation: clusters and bi-directionally associated domain pairs and explore their functional importance and phylogenetic conservation.

**Conclusion:**

Taking into account the order of domains, we have derived a novel picture of global protein organization. We found that all genomes have a higher than expected degree of clustering and more domain pairs in forward  and reverse orientation in different proteins relative to random graphs with identical degree distributions. While these features were statistically over-represented, they are still fairly rare. Looking in detail at the proteins involved, we found strong functional relationships within each cluster. In addition, the domains tended to be involved in protein-protein interaction and are able to function as independent structural units. A particularly striking example was the human Jak-STAT signalling pathway which makes use of a set of domains in a range of orders and orientations to provide nuanced signaling functionality. This illustrated the importance of functional and structural constraints (or lack thereof) on domain organisation.

## Background

One of the driving forces behind protein evolution is the duplication and shuffling of domains [1-3]. There are approximately 1500 known domain superfamilies that have been combined in different ways to form the protein repertoire (SCOP version 1.65, [4] and corroborated by [5]). Domains can be thought of as the building blocks of proteins. During evolution, pairs and groups of domains have joined to form multi-domain proteins. In many cases, these groups have then been preserved and duplicated to generate higher-order combinations [6-8]. Unravelling how this duplication and rearrangement has occurred allows us to understand the functional relationships between domains and determine how proteins have evolved. Structural and genome-scale studies have shown that pairs of domains that are adjacent on proteins are usually the result of a single recombination event which is preserved and duplicated as a unit. [9] found that N-C terminal order of any particular pair of domains is almost always preserved across protein space. That is, if the domain pair A-B is observed, B-A is unlikely to occur. [10] showed that this extends to triplets of domains and identified two and three domain patterns that are over represented. [11] showed that multi-domain architectures are almost always the result of a single recombination event with convergent evolution to generate a particular pattern of domains being rare. Further, structural analysis of the geometry of adjacent domains indicated that most domain pairs we observe next to each other on a protein have become joined once in evolution [12]. Recent studies have found higher rates of domain architecture re-invention, but the proportion of these cases is still low [13].

Some domains occur in multiple of different domain architectures. They are relatively rare, typically involved in protein-protein interactions [14] and most often located at the ends of proteins or as single domain proteins [15].

Previous global studies of domain organisation have used undirected graphs to represent the co-occurrence of domains within proteins [16-23]. These studies represented proteins as a graph with vertices corresponding to domains and edges linking domains that are found within one protein. This model views a protein as a "bag of domains" because all domains on the protein are linked irrespective of their order or relative positions. For example, [16] analysed the global properties of the network showing that the domain graph has small-world and scale-free topology and [17] used the domain graph representation to compare changes in domains and combinations between genomes. These analyses concentrated on the global properties of the network and used the "bag of domains" model which does not account for evolutionary changes manifested through re-ordering of domains. We set out to consider the functional and evolutionary importance of domain order by building a model that accounts for the relative position of domains on a protein. Given the importance of domain order as shown by structural and sequence-based studies (described above), incorporation of information about sequential domain arrangements may lead to novel insights into how proteins are organised.

**Figure 1:**
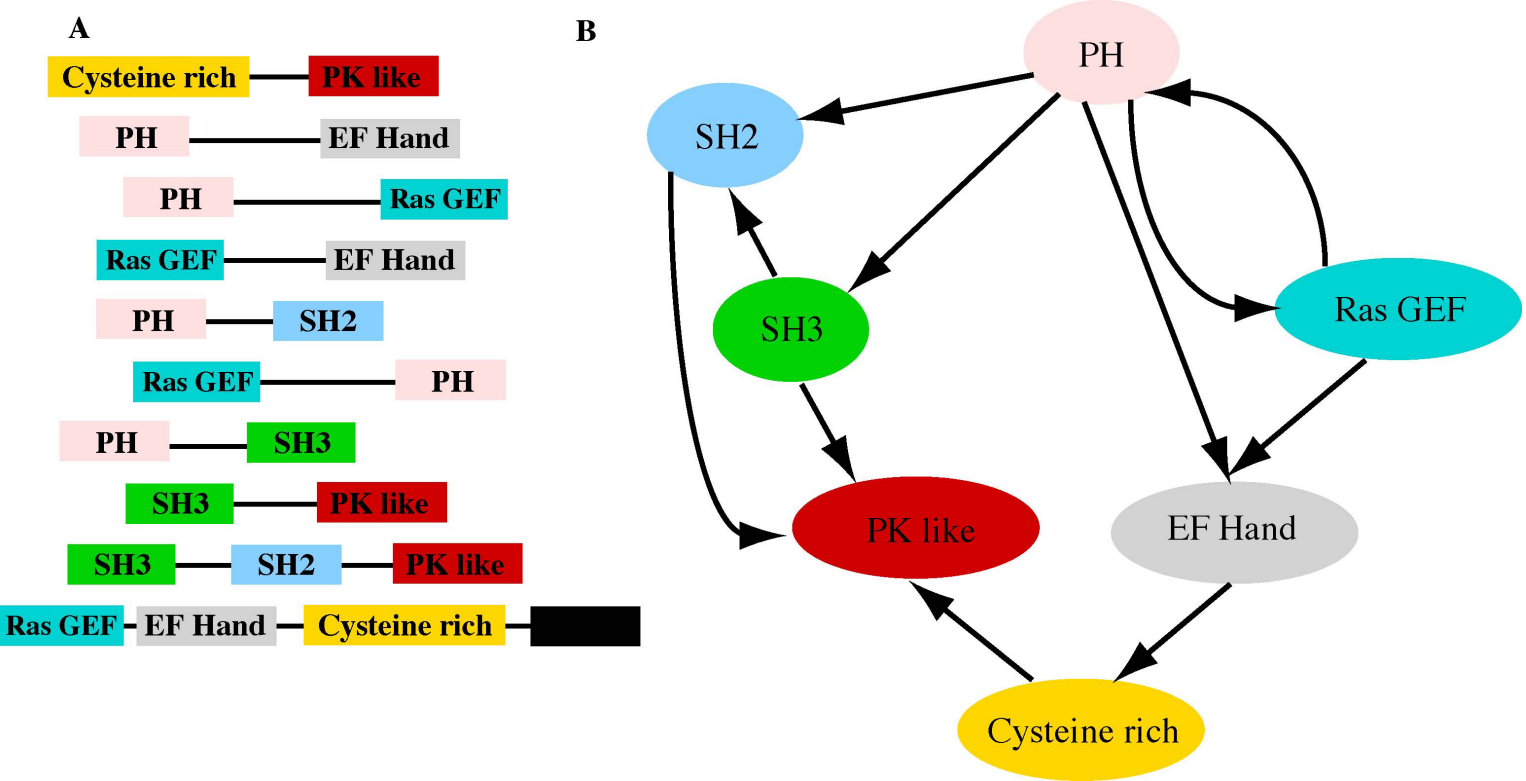
**Domain architectures and the directed domain graph**. A) Eight domain architectures are shown. Each coloured rectangle represents a domain superfamily. B) The graph corresponding to the eight architectures in (A). Ovals represent domain superfamilies (nodes in the graph) and edges indicate N-C terminal arrangement of domains within proteins.

We describe a directed domain graph that takes into account the sequential order of domains. To begin, we evaluate the global properties of the directed networks and compare them with random and previously studied examples. We then assess the domain graphs for individual genomes and phylogenetic groups. Aside from global properties, our analysis also identified cases of non-linear domain organisation. For example, domains form clusters that are highly inter-linked, in particular with links between pairs of neighbours [see Additional file 1] exemplified by Figure 1. While in general the linear order of domains is conserved, we identified groups of domains that have been shuffled during evolution so that they occur in a range of permutations across different proteins. This prompts a multitude of questions: why do these exceptional arrangements exist? is there something special about the functions or structures of domains that occur within such clusters? Are the non-linear arrangements specific to genomes or are they conserved in evolution? We consider 192 genomes across all three kingdoms of life and use domain/protein annotation to investigate the functional significance of these higher-order patterns of domain organisation. Finally, we assess the phyletic distribution of clusters in order to establish their evolutionary relationships. structures where domains are defined by to the Structural Classification of Proteins

## Results and discussion

### 0.1 Global features of domain organisation

We consider proteins in terms of their domains based on the SCOP database [4] superfamily definition of a domain. Domains were assigned to proteins using the SUPERFAMILY database (v1.65, [24]) predictions and include 192 completely sequenced genomes (19 archaea, 129 bacteria and 44 eukaryotes, [see Additional file 2]). These assignments are used to determine the sequential order of domains along a protein, termed the protein's domain architecture. We represented domain architectures as a directed graph with superfamilies as nodes and directed edges linking adjacent domains from the N- to C-terminus (Figure 1). We chose the SUPERFAMILY database because it represents domains that highly divergent but evolutionarily related.

This section presents the global properties of the directed domain graph, establishes its topology and compares it with the previously studied "bag-of-domains" model [16,17]. The properties we considered were: mean degree, degree distribution, mean clustering coefficient, characteristic path length and network density. We explain each network parameter, describe the values observed for the directed domain graph in comparison to random expectation and discuss their biological significance.

The degree distribution [see Additional file 1] of the directed domain graph follows a power-law: a small number of superfamilies have many neighbours, while the majority have only one or two. Networks with power-law degree distribution are described as having scale-free topology [25].

In order to assess the significance of the domain graph's global network properties, we compared the observed values with those expected at random. The randomly expected values were determined by calculating the network properties for 1000 random graphs. We were specifically interested in the network properties that are impacted by domain order: the clustering relationships of domains, the density and connectedness of the network.

In designing our randomisation strategy it was important to consider both the known properties of the graph and the parameters of interest. We knew that the domain graph was scale free. A simple randomisation approach that preserves only the number of nodes and edges generates a truly random graph, with a degree distribution that follow a Poisson distribution [25]. By definition, such random graphs will exhibit significantly different global properties from their scale-free counterparts, making comparisons between observed network properties and these random graphs meaningless.

**Figure 2:**
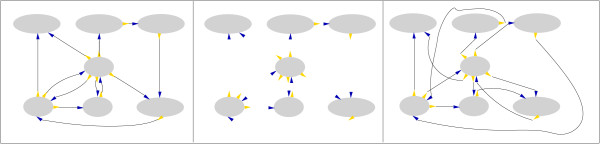
**Algorithm for generating random scale-free graphs with a fixed degree distribution**. (A) The algorithm takes the observed domain graph. The example shown here has incoming edges marked in blue and outgoing in yellow. (B) The in and out-degrees for each node are recorded while the connectivity is discarded. (C) The program randomly selects one node from each of the in- and out-degree lists. This pair of nodes is connected and removed from their respective lists. Through a series of iterations, the graph is assembled. If a selected pair of nodes is already present in the partially built graph, they are returned to the candidate lists and a second pair is randomly chosen. Towards the completion of the graph it is not uncommon for all possible new pairs to already be in the graph because the domain graph is scale-free with a small number of high-degree nodes. In this case, an edge is randomly selected from the partially built graph and the the inward links are exchanged. This procedure generates random graphs with identical degree distribution to the initial graph.

To overcome this problem, we generated random graphs with precisely the same degree distribution as the observed graph [26,27] (explained in Figure 2). This allowed us to assess the bidirectionality, clustering, density and connectedness of the network. We compared the clustering and density of nodes in the observed network to our random model. The mean clustering coefficient [see Additional file 1] is higher than expected at random and the characteristic path length [see Additional file 1] is longer (Table 1). This is consistent with small-world topology [28] and is also observed for the "bag-of-domains" model [16,17]. The graph has a lower density than would be expected at random meaning that a smaller fraction of possible pairs of nodes are directly/indirectly connected. This suggests that on a global level, domains have tended not to recombine with many partners that are also highly connected. Instead there are clear hubs, nodes that are highly connected which link to relatively poorly connected neighbours.

**Table 1:** Global properties of the domain architecture network.

**Property**	**Observed**	**Random (mean)**	**Stdev**	**Deviation from observed**
**Directed**				

Mean Degree	3.59	3.59	0	0
Mean Clustering Coefficient	0.14	0.05	0.004	+22.5*SD*
Characteristic Path Length	3.87	3.83	0.02	+2*SD*
Network density (% connected)	57.7	60.6	0.58	-5*SD*

**Undirected**				

Mean Degree	6.47	7.00	0	0
Mean Clustering Coefficient	0.23	0.08	0.007	+20.7*SD*
Characteristic Path Length	3.40	3.29	0.02	+5.5*SD*
Network density (% connected)	93.02	94.8	0.64	-2.8*SD*

The graph represents 6183 distinct domain architectures (from 262,481 proteins in 192 genomes) including 1224 different domain superfamilies. The mean values and standard deviations of each property are for 1000 random graphs, each generated to have the same in- and out-degree as the domain organisation graph (Figure 2). The characteristic paths lengths for directed and undirected graphs are numerically quite similar. However, these numbers cannot be directly compared because the proportion of nodes that are reachable (and hence included in the path length calculation) is much higher in the undirected graph (61% for the directed compared to 95% for the undirected).

**Figure 3:**
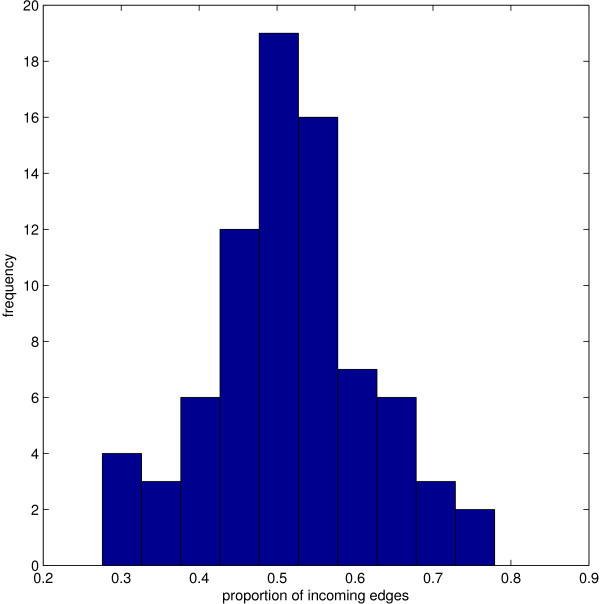
**In- and Out-degree distribution of the domain graph**.  Frequency histogram showing the number of incoming edges divided by the total number of edges. This shows the mean 0.502, standard deviation 0.07.

New to our directed model of domain organisation is the notion of inward and outward edges; as a result, we have two degree distributions (incoming and outgoing). Both the in- and out-degree obey a power-law distribution. Highly connected nodes (i.e. those with more than 50 edges) have in- and out-degrees that are correlated so that any particular node has approximately equal numbers (mean 0.52, standard deviation 0.07) of inward and outward links (Figure 3). This shows that domains combine with partner domains equally on their N and C sides. Note that the incoming and outgoing links generally come from or go to **different **domain superfamilies. There are only a small number of cases where one pair of domains has links in both directions, these are discussed below.

### 0.2 Genome domain graphs

The domain graph above included domain architectures from all proteins in our set of 192 genomes, providing a broad picture of protein organization. By including proteins from different genomes in a single graph, the domains represented may not all interact physically or during evolution. In order focus on evolutionary and functional features of domain organisation, we considered the global network parameters for the graphs of domains from individual genomes and phylogenetic groups of genomes. We calculated the graph parameters expected at random separately for each genome or group through 1000 randomisation of each graph.

**Figure 4:**
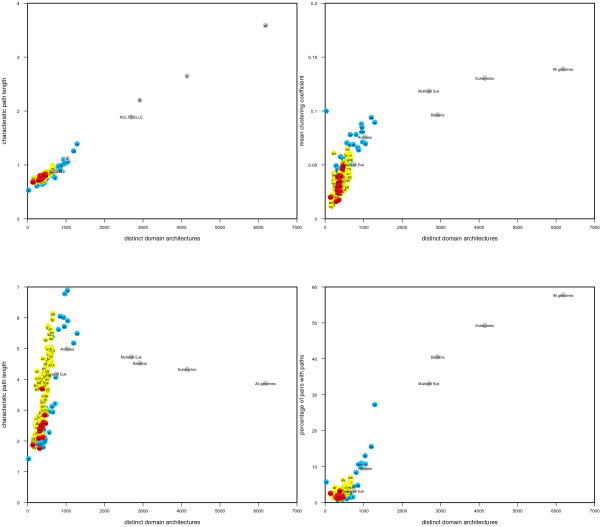
**Distinct domain architectures compared to genome graph properties**. a) Mean Degree b) Mean Clustering Coefficient c) Characeristic Path Length d) Percentage of pairs with paths. Each genome is represented by one point (labelled with its abbreviation used in the SUPERFAMILY database). Archaea are coloured red, bacteria yellow, eukaryotes blue and groups of genomes (e.g. all archaea) are shown in grey. There is a strong correlation between the mean degree and number of distinct architectures (correlation coefficient 0.99). Clustering coefficient (0.85), characteristic path length (0.73) and percentage of connected pairs (0.88) are also correlated, though more weakly.

Restricting the domain set to single or groups of genomes does not affect the global graph topology compared to random; all are scale-free and small-world. However, there is considerable variation in the network parameters (see Table 2 [see Additional file 2 for the full list of 192 genomes]). The differences can in part be attributed to the number of domains and domain architectures within each genome. In particular, the mean degree correlates strongly with the number of distinct domain architectures (Figure 4). The overall node connectivity, quantified through clustering coefficient and characteristic path length also correlate with the number of distinct domain architectures (though more weakly). This suggests that the level of small-world-ness we observe in different genomes is largely, but not solely, a function of the number of proteins or domain architectures present. The clustering coefficient and percentage of pairs with a direct path for the three kingdoms are broadly grouped (Figures 4B and 4D), suggesting that the characteristics of a genome's protein repertoire are also important for determining the level of connectivity of the domain graph.

**Table 2:** Global properties of the genome domain architecture network

Genome/s	Domains	Degree	Clustering Coefficient	Density	Path Length
all genomes	1070	3.59 (3.59)	0.1390 (21.9)	57.74 (5.0)	3.87 (2.0)
archaea	424	1.12 (1.12)	0.0760 (19.1)	9.57 (3.2)	4.99 (1.1)
bacteria	794	2.20 (2.20)	0.0965 (18.5)	40.57 (2.5)	4.53 (2.6)
eukaryotes	890	2.65 (2.65)	0.1306 (22.8)	49.32 (4.9)	4.33 (6.3)
unicellular eukaryotes	341	0.88 (0.88)	0.0504 (18.9)	3.23 (1.1)	4.18 (1.1)
multicellular eukaryotes	739	1.89 (1.89)	0.1186 (24.0)	33.12 (6.9)	4.73 (1.5)

*Saccharomyces cerevisiae*	193	0.72 (0.72)	0.0415 (16.0)	1.09 (1.6)	2.32 (1.3)
*Escherichia coli*	343	0.86 (0.86)	0.0389 (15.7)	4.26 (0.3)	5.53 (0.5)
*Caenorhabditis elegans*	317	0.81 (0.81)	0.0592 (21.0)	2.65 (0.9)	5.38 (0.4)
*Homo sapiens *22.34d	389	0.99 (0.99)	0.0639 (21.5)	10.62 (1.0)	7.30 (1.3)
*Mus musculus *22.32b	416	1.02 (1.02)	0.0807 (25.7)	10.66 (0.7)	7.80 (1.4)

For each genome or phylogenetic group of genomes, we calculated the number of domains, distinct domain architectures, mean degree, mean clustering coefficient, network density (as a percentage of pairs that are connected) and the characteristic path length (each of these properties is defined [see Additional file 1]). The number of standard deviations between the randomised and observed graphs is included in parentheses. The parameters for all 192 genomes considered are provided [see Additional file 2].

**Figure 5:**
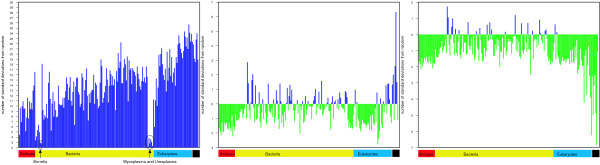
**Standard deviations between observed and random for all genomes**. a) Clustering coefficient b) Path length c) Density. Plots show for each genome (x-axis) the number of standard deviations (based on 1000 randomisations of each graph while preserving scale free topology) between the observed and expected values (y-axis). For example, a bar that stops at -3 indicates the observed value was three standard deviations below the randomly expected value. Positive values are shown in blue, negative in green. Genomes have been grouped by kingdom as indicated by the red, yellow and blue regions below the x-axis. The black region marks the graphs that are combinations of genomes in the following order: all genomes, archaea, bacteria, eukaryotes, multicellular eukaryotes and unicellular eukaryotes. The mean degree was excluded from this analysis because it was intentionally fixed for the randomisation experiments.

An alternative approach to comparing network parameters across genomes is to consider the extent to which each individual organism differs from the expected values calculated from randomisation experiments. We assessed the difference between the observed parameters and the mean of the randomised trials by calculating their difference in terms of the number of standard deviations. Figure 5 plots the number of standard deviations between the observed and randomly expected values for each genome. The observed average clustering coefficient (Figure 5A) is consistently more than two standard deviations above the expected value for all genomes. This indicates that a high degree of clustering of domains occurs across all genomes. Three groups of bacteria, *Borrelia, Mycoplasma *and *Ureaplasma*, stand out as having a relatively small difference between the observed and expected clustering coefficient. This could be related to the fact that these organisms are all parasites with small genomes.

In contrast, the percentage of connected nodes and average path length are not consistently higher or lower than expected. The percentage of connected domains for the majority of genomes is slightly lower (0.5 and 2 standard deviations) than expected; while around 20 bacterial species have values slightly higher than expected. The average path lengths present a more complicated picture. For the all-genome graph, the observed path length is longer than expected. However, for all the archaea, a large proportion of eukaryotes and around half of the bacteria, the average path length is slightly lower than expected. For the most part the single-genome values fall within two standard deviations of the mean and may not be significant. This suggests that average path lengths are no different from the values expected at random and therefore not influenced or controlled by functional or evolutionary constraints.

Section 1 investigates this variability between genomes further by looking on a case-by-case basis at the functional significance of these clusters and their phylogenetic distribution.

### 0.3 Bi-directional paths

[29] and 9 demonstrated that N-C terminal domain order is generally conserved across proteins. That is, if domain A is found N-terminal to domain B, the reverse combination (BA) tends not to exist. [12] and [30] looked in detail at the structures of proteins that contradict this rule such that one protein has domain combination AB while a second contains BA. They found that in general the relative structural orientation of domains A and B was different in the forward compared to the reverse case and the proteins have different functions. The clusters above illustrate that there are cases where pairs of domains are observed in both orientations; we call these bi-directional paths.

From the genome level domain graphs, we identified all bi-directional paths (a selection of genomes are listed in Table 3). We found that while the percentage of adjacent domain pairs found in both forward and reverse orientations is small (between around 3% and 6%), these values are significantly higher than expected at random. This means that bidirectional links are over-represented in genomes. Note that this does not contradict previous studies because we are concerned with number of different bidirectionally oriented domain pairs regardless of the prevalence of these pairs within the protein repertoire. In contrast, [29] counted the number of any given pair of occurrences within the protein repertoire and observed that bidirectionally linked domain pairs are rare. The domain pairs found in both orientations in human are shown in Table 4). It is interesting to note that they include signal transduction and protein interaction domains.

**Table 3:** Bi-directional paths

Genome	% bidirectional links
*Caenorhabditis elegans*	4.9 (11.0)
*Escherichia coli*	3.1 (6.5)
*Homo sapiens*	6.3 (14.4)
*Mus musculus*	5.5 (14.5)
*Saccharomyces cerevisiae*	6.2 (10.4)

The number of pairs of nodes with bi-directional paths connecting them. Within the set, some are the result of a single domain architecture with domains arranged ABA while others occur in multiple proteins with one domain architecture AB and another BA. The percentage of adjacent domain pairs with bidirectional links is given followed by the number of standard deviations between the randomised and observed domain graphs. We see a significant difference in all cases (p < 0.001).

**Table 4:** Human bidirectionally-linked domains

**Domain 1**	**Domain 2**
GlnB-like	P-loop containing nucleoside triphosphate hydrolases
Phosphoglycerate kinase	P-loop containing nucleoside triphosphate hydrolases
Concanavalin A-like lectinsglucanases	EGFLaminin
Elafin-like	BPTI-like
TPR-like	Ankyrin repeat
ARM repeat	SAM/Pointed domain
EF-hand	P-loop containing nucleoside triphosphate hydrolases
p53-like transcription factors	SH2 domain
Serine proteinase inhibitor lekti	Ovomucoid/PCI-1 like inhibitors
RNA-binding domain, RBD	CCCH zinc finger
"Winged helix" DNA-binding domain	PH domain-like
C2H2 and C2HC zinc fingers	Microbial and mitochondrial ADK, insert "zinc finger" domain

#### 1 Local features of domain organisation

The global analysis described above highlighted features of domain organisation occurring more often than expected at random that cannot be explained by a simple linear model of domain combination evolution. First, the level of clustering within the directed domain graphs is higher than expected at random. That means that some groups of domains have recombined in multiple different ways. Second, some domain pairs are found in two different N-C terminal orders, and this occurs more often than expected at random. That is, domain A is sometimes followed and other times preceded by B, providing evidence of functional but not evolutionary links. This section discuss these features locally, using functional annotation to investigate why they occur and how they are distributed across phylogenetic groups.

#### 1.1 Function of domain clusters

One of the most pronounced features of the domain graph is the higher than expected mean clustering coefficient. For the complete domain graph, the mean clustering coefficient is 22.5 standard deviations above that of the random graphs. In comparison, the characteristic path length and network density are only +2 and -5 standard deviations from the random mean. A high mean clustering coefficient indicates that the domain graph includes groups of nodes whose neighbours are interlinked. In the context of domain organisation, this means that there are groups of promiscuous domains that occur in multiple different combinations.

To investigate the functional and evolutionary features of these clusters, we extracted groups of domains with inter-linking neighbours from genome level domain organisation graphs for 192 completely sequenced genomes. This gave us a list of proteins present in each cluster. In order to identify functional relationships, we used the KEGG pathway database [31] and Gene Ontology (GO) annotation. This allowed us to extract clusters of domains with every node represented in a particular functional category. We focused on GO molecular function and biological process categories with between 5 and 500 member proteins (in order to exclude both overly specific and very large, non-specific functional groups). We found that for every cluster, all nodes belong to at least one common GO category; significantly more than expected by chance (p < 0.001 [see Additional file 3]). Statistical significance was assessed by comparing the proportion of randomly selected groups of domains that belong to a common functional class with the observed proportion. Random groups were sampled from the entire domain graph and chosen to be the same size as each observed cluster; randomisation was carried out 1,000 times per cluster.

**Figure 6:**
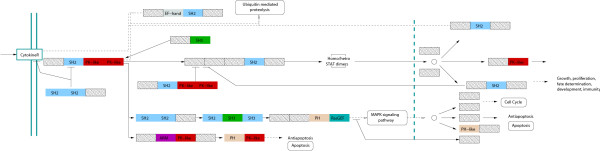
**Eukaryotic signal transduction: The Jak-STAT signalling pathway.** Proteins are represented by their domain architectures shown as a series of rectangles, one for each domain. The labelled domains found in the Homo sapiens graph cluster (Figure 1) include SH2 (blue), PK-like (red), PH (pink), SH3 (green), RasGEF (turquoise), ARM (magenta). The arrows illustrate the Jak-STAT signalling pathway (derived from the KEGG database).

For example, considering the clusters shown in Figure 6, we find 19 different GO categories that are common within one or more clusters. These include: cell-cell signaling, identical protein binding, induction of apoptosis and receptor binding. (A complete list is provided [see Additional file 3].)

**Figure 7:**
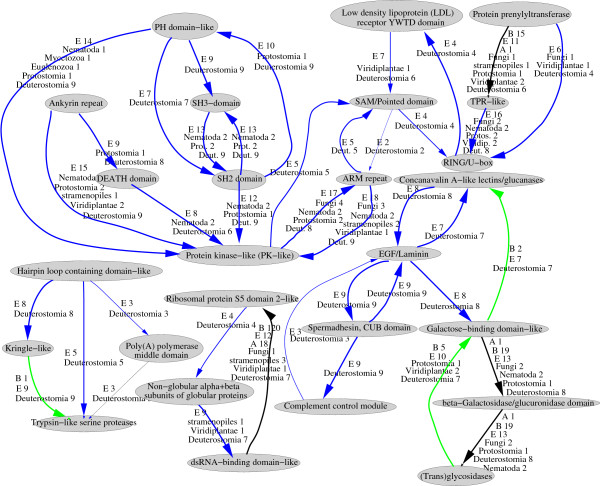
**Homo sapiens clustered domains indicating phyletic distribution.** Domains with a clustering coefficient greater than zero are shown. The phyletic distribution of each domain pair is indicated by the edge width (proportional to the number of genomes in which it is observed), the colour of the edge (blue for eukaryote specific, green for present in eukaryotes and bacteria and black for present in all three kingdoms) and the label listing the number of genomes within each group possessing the domain pair.

The Jak-STAT signalling pathway (from the KEGG database, [31]) includes domains that form a cluster. Figure 7 shows the pathway with each protein represented by its domain architecture (derived from the SUPERFAMILY database, [24]). A small number of domains including: SH2, SH3, PH-like and PK-like, recur in multiple combinations. Signal transduction is known to involve highly modular proteins; made up of a relatively small number of components that have mixed-and-matched to generate all necessary functions [32]. By re-using domains in different contexts, a relatively small domain repertoire can produce a range of wirings within the cell.

The clusters we describe are an extreme form of re-use, because not only have individual domains recombined with multiple partners, but their partners have recombined with each-other. Aside from signal-transduction domains, the two central human clusters (shown in figure 7) also include protein-interaction domains; for example, the Ankyrin repeat domain. These clusters are examples where the proteins involved make use of the protein interaction domains in different combinations and with other partners to diversify or specialise their functions.

This suggests that functional and structural constraints (or lack there-of) can lead to exceptional arrangements of domains. For instance, multi-domain structures that are essentially beads-on-a-string with no fixed interface between domains are more likely to be functional in multiple orientations than their tightly (structurally) inter-linked counterparts. Many of the domains we observe within our clusters can function independently of their neighbours.

#### 1.2 Phyletic patterns of human domain clusters

The clusters that we observed in *Homo sapiens *are almost exclusively eukaryote-specific (shown in Figure 6) and for the most part the domains that occur within the clusters are themselves found only in eukaryotes. Even for the domains that are found in bacteria, the particular combinations we observe in clusters are peculiar to eukaryotes.

The great majority of human clusters are also found in Chimpanzee and almost all of these are present in mouse and rat. Looking at more distantly related Chordate genomes the conservation declines rapidly with only the central cluster common to chicken and many links missing in *Xenopus tropicalis*, *Fugu rubripes*, Danio rerio and *Ciona intestinalis*. If we look to even more distantly related species, for example, *Saccharomyces cerevisiae*, none of the clusters are conserved.

## Conclusion

The arrangement of domains within proteins has been studied previously using a graph representation where nodes are domains and edges join domains observed within a single protein. A shortcoming of this representation is that it does not take into account the N to C terminal arrangement of domains on the protein. We have developed a directed graph model of domain organisation that considers order and relative N to C terminal position. By investigating the global properties of the network, we have shown that domain clustering occurs significantly more often than expected at random.

Considering each genome in isolation we found that the high degree of clustering observed for the multi-genome dataset also holds for each genome individually. However, the characteristic path length and percentage of connected nodes are not very different from the randomly expected values. These findings suggest that the domain organisation of individual genomes varies but all show a higher than expected degree of clustering.

Focusing in detail on domain clusters, we identified functional constraints that make this arrangement highly preferable to the organism. Clusters in human are almost exclusively eukaryote-specific and have roles in signal transduction and protein-protein interaction.

Finally, we observe pairs of domains found in forward and reverse orientation in different proteins more often that would be expected at random. While previous work has shown that this phenomenon is rare in terms of the number of occurrences in proteins, we see the opposite trend for the existence of such domain pairs. The function of the domains that occur in both orientations are similar to those found in clusters suggesting a common underlying functional or structural cause.

## Authors' contributions

SKK designed the study, developed and implemented algorithms and drafted the manuscript. SAT assisted with designing the study and analysing the results

## Supplementary Material

Additional file 1 **Network properties. **The cartoons above illustrate the network properties calculated for the directed domain graph. Click here for file

Additional file 2 **Global properties of the genome domain architecture network.** For each genome or phylogenetic group of genomes, we calculated the number of domains, distinct domain architectures, mean degree, mean clustering coefficient, network density (as a percentage of pairs that are connected) and the characteristic path length (each of these properties is defined [see Additional file 1]). The mean values and standard deviations of each property shown in parentheses are for 1000 random graphs, generated to have the same in- and out-degree as the domain organisation graph for each group of genomes (the algorithm for generating these graphs is described in Additional File 1).
Click here for file

Additional file 3 **Function of domain clusters.** The functional relationships between domains in each cluster were assessed using GO annotation. We found that all clusters with a clustering coefficient greater than zero had at least one GO category in common. Permutation analysis, taking into account the size of each GO category, indicated that this is statistically significant (p < 0.001). This table lists, for each cluster with a clustering coefficient greater than 0 (identified by the central node), the common GO category to which they belong and the number of nodes that are included in the cluster. Click here for file

## References

[B1] Brenner SE, Hubbard T, Murzin A, Chothia C (1995). Gene duplications in the H. influenzae genome. Nature.

[B2] Teichmann SA, Park J, Chothia C (1998). Structural assignments to the Mycoplasma genitalium proteins show extensive gene duplications and domain rearrangements. Proc Natl Acad Sci USA.

[B3] Apic G, Gough J, Teichmann SA (2001). An insight into domain combinations. Bioinformatics.

[B4] Murzin AG, Brenner SE, Hubbard T, Chothia C (1995). SCOP: a structural classification of proteins database for the investigation of sequences and structures. J Mol Biol.

[B5] Orengo C, Thornton J (2005). Protein families and their evolution-a structural perspective. Annual Review of Biochemistry.

[B6] Koonin EV, Wolf YI, Karev GP (2002). The structure of the protein universe and genome evolution. Nature.

[B7] Muller A, MacCallum RM, Sternberg MJE (2002). Structural characterization of the human proteome. Genome Res.

[B8] Gerrard DT, Bornberg-Bauer E (2003). doMosaic – Analysis of the mosaic-like domain arrangements in proteins. Informatica.

[B9] Apic G, Huber W, Teichmann SA (2003). Multi-domain protein families and domain pairs: comparison with known structures and a random model of domain recombination. J Struct Funct Genomics.

[B10] Vogel C, Berzuini C, Bashton M, Gough J, Teichmann S (2004). Supra-domains: evolutionary units larger than single protein domains. J Mol Biol.

[B11] Gough J (2005). Convergent evolution of domain architectures (is rare). Bioinformatics.

[B12] Bashton M, Chothia C (2002). The geometry of domain combination in proteins. J Mol Biol.

[B13] Forslund K, Henricson A, Hollich V, Sonnhammer EL (2008). Domain tree-based analysis of protein architecture evolution. Mol Biol Evol.

[B14] Basu MK, Carmel L, Rogozin IB, Koonin EV (2008). Evolution of protein domain promiscuity in eukaryotes. Genome Res.

[B15] Weiner JBBE, Moore AD (2008). Just how versatile are domains?. BMC Evol Biol.

[B16] Wuchty S (2001). Scale-free behavior in protein domain networks. Mol Biol Evol.

[B17] Ye Y, Godzik A (2004). Comparative analysis of protein domain organization. Genome Res.

[B18] Weiner J, Beaussart F, Bornberg-Bauer E (2006). Domain deletions and substitutions in the modular protein evolution. FEBS J.

[B19] Przytycka T, Davis G, Song N, Durand D (2006). Graph theoretical insights into evolution of multidomain proteins. J Comput Biol.

[B20] Wuchty S, Almaas E (2005). Evolutionary cores of domain co-occurrence networks. BMC Evol Biol.

[B21] Cohen-Gihon I, Nussinov R, Sharan R (2007). Comprehensive analysis of co-occurring domain sets in yeast proteins. BMC Genomics.

[B22] Qian J, Luscombe NM, Gerstein M (2001). Protein family and fold occurrence in genomes: power-law behaviour and evolutionary model. J Mol Biol.

[B23] Dokholyan NV, Shakhnovich B, Shakhnovich EI (2002). Expanding protein universe and its origin from the biological Big Bang. Proc Natl Acad Sci USA.

[B24] Gough J, Karplus K, Hughey R, Chothia C (2001). Assignment of homology to genome sequences using a library of hidden Markov models that represent all proteins of known structure. J Mol Biol.

[B25] Barabasi A, Albert R (1999). Emergence of scaling in random networks. Science.

[B26] Newman ME, Strogatz SH, Watts DJ (2001). Random graphs with arbitrary degree distributions and their applications. Physical review E, Statistical, nonlinear, and soft matter physics.

[B27] Bollobas B, Borgs C, Chayes J, Riordan O (2003). Directed scale-free graphs. Proceedings of the fourteenth annual ACM-SIAM symposium on Discrete algorithms.

[B28] Watts DJ, Strogatz SH (1998). Collective dynamics of 'small-world' networks. Nature.

[B29] Apic G, Gough J, Teichmann SA (2001). Domain combinations in archaeal, eubacterial and eukaryotic proteomes. J Mol Biol.

[B30] Todd AE, Orengo CA, Thornton JM (2001). Evolution of function in protein superfamilies, from a structural perspective. J Mol Biol.

[B31] Kanehisa M, Goto S (2000). KEGG: Kyoto Encyclopedia of Genes and Genomes. Nucleic Acids Res.

[B32] Pawson T, Nash P (2003). Assembly of cell regulatory systems through protein interaction domains. Science.

