# Supplementary figures and images for: Protein domain organisation: adding order
Source: BMC Bioinformatics. 2009 Jan 29;10:39. doi: 10.1186/1471-2105-10-39 (PMC2657131; doi:10.1186/1471-2105-10-39)

**A. Degree**

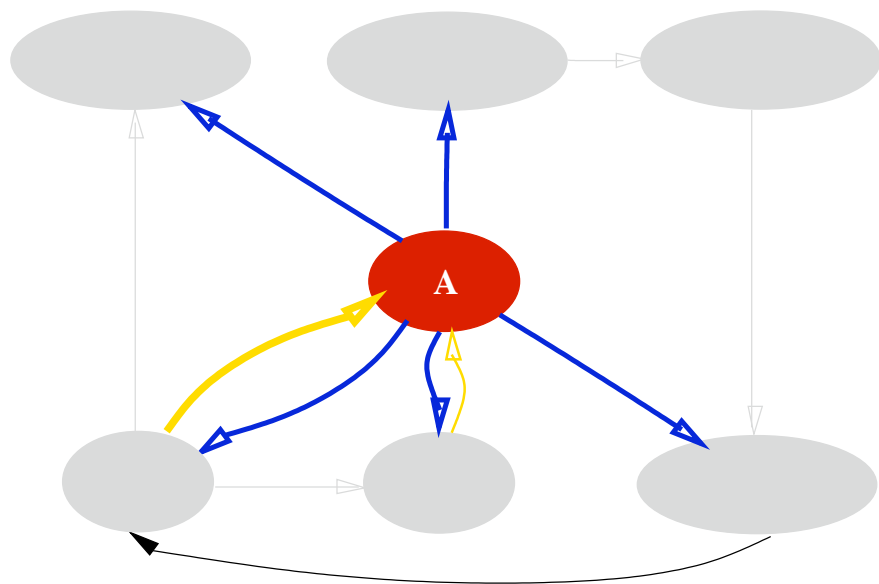

**B. Clustering Coefficient**

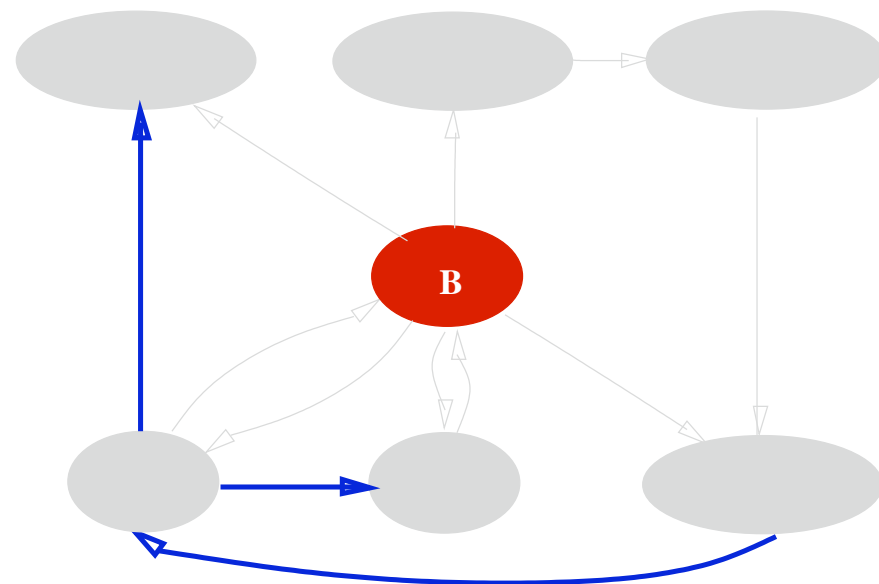

**C. Path Length**

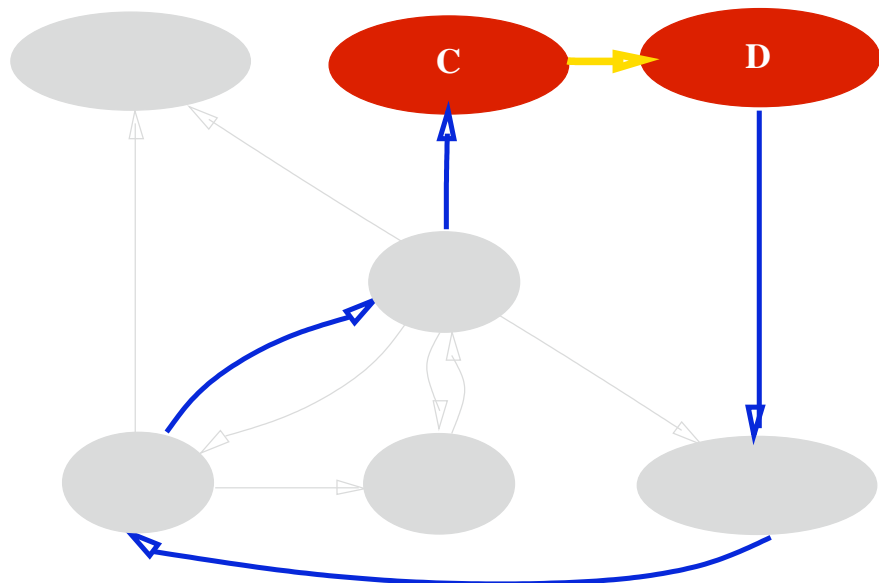

**D. Network density**

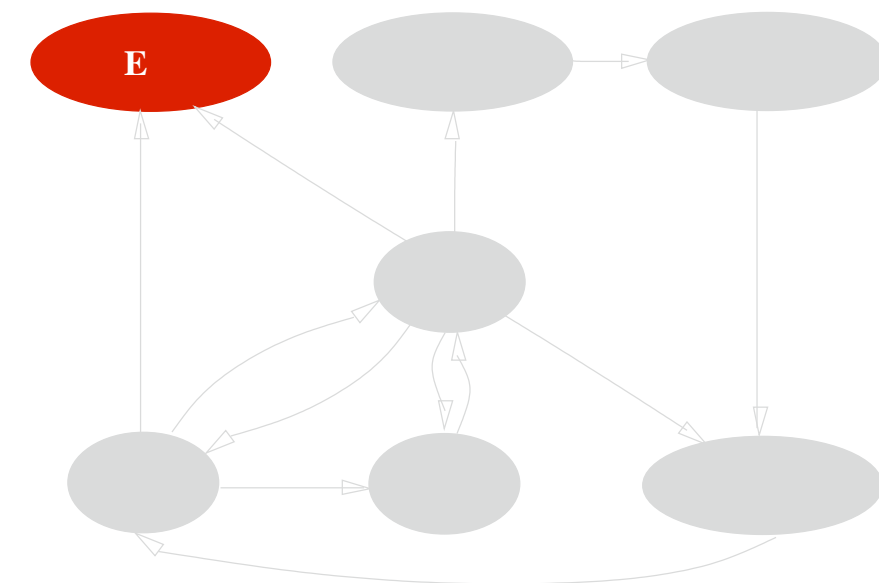

Supplement: Additional file 1 — Network properties. The cartoons above illustrate the network properties calculated for the directed domain graph. [file 1471-2105-10-39-S1.pdf]
