# Supplementary material for: Protein domain organisation: adding order
Source: BMC Bioinformatics. 2009 Jan 29;10:39. doi: 10.1186/1471-2105-10-39 (PMC2657131; doi:10.1186/1471-2105-10-39)
Supplement: Additional file 2 — Global properties of the genome domain architecture network. For each genome or phylogenetic group of genomes, we calculated the number of domains, distinct domain architectures, mean degree, mean clustering coefficient, network density (as a percentage of pairs that are connected) and the characteristic path length (each of these properties is defined [see Additional file 1]). The mean values and standard deviations of each property shown in parentheses are for 1000 random graphs, generated to have the same in- and out-degree as the domain organisation graph for each group of genomes (the algorithm for generating these graphs is described in Additional File 1). [file 1471-2105-10-39-S2.pdf]

Genome/s	Domains	Degree	Clustering Coefficient	Density	Path Length
all genomes	1070	3.59	0.1390 (0.0494, 0.0041)	57.74 (60.64, 0.58)	3.87 (3.83, 0.02)
archaea	424	1.12	0.0760 (0.0091, 0.0035)	9.57 (14.33, 1.50)	4.99 (5.36, 0.33)
bacteria	794	2.20	0.0965 (0.0282, 0.0037)	40.57 (43.02, 0.97)	4.53 (4.40, 0.05)
eukaryotes	890	2.65	0.1306 (0.0349, 0.0042)	49.32 (53.13, 0.78)	4.33 (4.14, 0.03)
unicellular eukaryotes	341	0.88	0.0504 (0.0031, 0.0025)	3.23 (4.44, 1.09)	4.18 (5.03, 0.78)
multicellular eukaryotes	739	1.89	0.1186 (0.0201, 0.0041)	33.12 (40.67, 1.10)	4.73 (4.64, 0.06)
Aquifex aeolicus VF5	218	0.76	0.0237 (0.0034, 0.0032)	1.90 (3.01, 0.70)	2.83 (3.55, 0.55)
Acinetobacter sp. ADP1	276	0.79	0.0353 (0.0020, 0.0022)	2.59 (2.69, 0.72)	4.51 (4.13, 0.71)
Propionibacterium acnes KPA171202	222	0.74	0.0269 (0.0015, 0.0020)	1.42 (1.91, 0.53)	2.68 (3.38, 0.68)
Archaeoglobus fulgidus DSM 4304	208	0.81	0.0394 (0.0031, 0.0028)	1.75 (4.26, 1.19)	2.60 (4.32, 0.77)
Anopheles gambiae 22.2b	385	0.91	0.0663 (0.0032, 0.0027)	4.80 (6.71, 1.46)	6.05 (6.10, 0.80)
Candida albicans	191	0.66	0.0431 (0.0008, 0.0020)	0.72 (1.06, 0.20)	1.80 (2.44, 0.44)
Aspergillus nidulans 1 r3.1	246	0.79	0.0399 (0.0024, 0.0027)	2.51 (3.14, 0.83)	4.02 (4.27, 0.77)
Aeropyrum pernix K1	140	0.72	0.0161 (0.0024, 0.0031)	1.49 (2.56, 0.64)	2.09 (3.00, 0.55)
Arabidopsis thaliana 5	338	0.92	0.0689 (0.0055, 0.0033)	4.46 (6.95, 1.25)	4.07 (4.98, 0.55)
Agrobacterium tumefaciens C58	118	0.68	0.0611 (0.0013, 0.0033)	1.43 (1.55, 0.28)	2.05 (2.19, 0.37)
Mycobacterium avium ssp. *paratuberculosis k10*	267	0.80	0.0417 (0.0025, 0.0026)	2.36 (3.26, 0.87)	4.01 (4.30, 0.75)
Bacillus anthracis Ames	317	0.85	0.0366 (0.0038, 0.0025)	4.28 (4.18, 1.07)	4.80 (4.52, 0.67)
Borrelia burgdorferi B31	117	0.74	0.0134 (0.0028, 0.0036)	2.42 (2.98, 0.64)	2.54 (2.79, 0.45)
Bacillus cereus ATCC 14579	310	0.87	0.0420 (0.0037, 0.0027)	4.34 (4.34, 1.17)	4.78 (4.71, 0.73)
Bdellovibrio bacteriovorus HD100	236	0.79	0.0510 (0.0028, 0.0028)	2.06 (2.81, 0.74)	3.71 (3.67, 0.63)
Bordetella pertussis Tohama I	267	0.82	0.0531 (0.0030, 0.0029)	2.71 (4.04, 1.03)	3.89 (4.47, 0.74)
Candidatus Blochmannia floridanus	148	0.70	0.0337 (0.0019, 0.0028)	1.59 (2.16, 0.44)	2.26 (2.70, 0.42)
Bacillus halodurans C-125	306	0.83	0.0294 (0.0033, 0.0024)	4.76 (4.01, 1.06)	4.85 (4.54, 0.73)
Bradyrhizobium japonicum USDA 110	303	0.93	0.0604 (0.0038, 0.0029)	5.53 (7.54, 1.85)	5.94 (5.86, 0.85)
Bifidobacterium longum NCC2705	175	0.72	0.0276 (0.0014, 0.0024)	1.19 (1.69, 0.40)	2.13 (2.84, 0.57)
Brucella melitensis 16M	254	0.81	0.0402 (0.0025, 0.0026)	2.86 (3.46, 0.96)	4.11 (4.47, 0.83)
Bordetella bronchiseptica RB50	289	0.87	0.0591 (0.0035, 0.0030)	3.55 (5.37, 1.33)	4.67 (4.94, 0.73)
Bordetella parapertussis 12822	286	0.85	0.0596 (0.0031, 0.0028)	3.39 (4.64, 1.15)	4.61 (4.79, 0.73)
Bartonella quintana Toulouse	179	0.73	0.0260 (0.0020, 0.0026)	2.57 (2.38, 0.54)	3.90 (3.20, 0.55)
Brucella suis 1330	250	0.80	0.0336 (0.0022, 0.0024)	2.49 (2.74, 0.74)	4.00 (3.99, 0.76)
Bacillus subtilis ssp. *subtilis* 168	316	0.84	0.0359 (0.0028, 0.0024)	4.80 (3.76, 1.14)	5.46 (4.80, 0.84)
Bacteroides thetaiotaomicron VPI-5482	263	0.83	0.0486 (0.0026, 0.0026)	2.70 (3.77, 0.98)	4.21 (4.54, 0.76)
Buchnera aphidicola Bp	144	0.71	0.0421 (0.0016, 0.0028)	2.79 (2.22, 0.54)	3.81 (2.97, 0.59)
Chlamydophila caviae GPIC	153	0.75	0.0358 (0.0024, 0.0032)	2.95 (2.65, 0.62)	4.03 (3.12, 0.55)
Clostridium acetobutylicum ATCC 824	282	0.82	0.0379 (0.0026, 0.0024)	2.87 (3.10, 0.94)	4.28 (4.34, 0.81)
Caulobacter crescentus CB15	271	0.83	0.0377 (0.0027, 0.0025)	2.47 (3.76, 1.09)	3.69 (4.63, 0.87)

Corynebacterium diphtheriae NCTC 13129	230	0.73	0.0338 (0.0012, 0.0020)	0.94 (1.36, 0.36)	2.18 (3.05, 0.66)
Caenorhabditis elegans WS123	317	0.81	0.0592 (0.0025, 0.0027)	2.65 (3.60, 1.06)	5.38 (5.03, 0.92)
Corynebacterium glutamicum ATCC 13032	235	0.78	0.0396 (0.0021, 0.0025)	1.55 (2.61, 0.78)	3.00 (3.84, 0.74)
Chlorobium tepidum TLS	235	0.77	0.0235 (0.0025, 0.0023)	1.83 (3.01, 0.73)	2.87 (3.83, 0.60)
Ciona intestinalis 1.0	380	1.10	0.0880 (0.0051, 0.0034)	10.98 (17.83, 1.62)	6.01 (6.20, 0.33)
Campylobacter jejuni ssp. *jejuni* NCTC 11168	196	0.74	0.0288 (0.0022, 0.0026)	1.87 (2.39, 0.60)	2.99 (3.33, 0.59)
Chlamydia muridarum	136	0.76	0.0364 (0.0039, 0.0044)	2.64 (3.30, 0.76)	2.73 (3.07, 0.49)
Corynebacterium efficiens YS-314	216	0.77	0.0323 (0.0018, 0.0024)	1.75 (2.64, 0.72)	3.30 (4.01, 0.78)
Chlamydophila pneumoniae J138	142	0.74	0.0391 (0.0026, 0.0035)	2.00 (2.67, 0.58)	2.44 (2.92, 0.48)
Synechocystis sp. PCC 6803	262	0.77	0.0455 (0.0019, 0.0023)	1.40 (1.97, 0.54)	3.49 (3.61, 0.71)
Chlamydia trachomatis D/UW-3/CX	134	0.76	0.0399 (0.0031, 0.0040)	2.24 (3.07, 0.75)	2.53 (3.03, 0.52)
Clostridium perfringens 13	266	0.80	0.0329 (0.0026, 0.0024)	2.13 (2.72, 0.72)	3.01 (3.77, 0.63)
Caenorhabditis briggsae Aug03	309	0.82	0.0781 (0.0025, 0.0032)	1.41 (3.56, 0.98)	2.94 (4.90, 0.83)
Coxiella burnetii RSA 493	229	0.79	0.0319 (0.0033, 0.0029)	3.83 (3.51, 0.81)	4.68 (3.87, 0.58)
Chlamydomonas reinhardtii 2.0	226	0.80	0.0561 (0.0035, 0.0032)	1.73 (3.33, 0.92)	2.97 (3.93, 0.71)
Danio rerio 22.3b	459	1.26	0.0940 (0.0081, 0.0036)	15.60 (24.15, 1.37)	5.18 (5.39, 0.16)
Debaromyces hansenii	197	0.67	0.0371 (0.0011, 0.0021)	0.86 (1.24, 0.25)	2.00 (2.60, 0.44)
Drosophila melanogaster 3.2	264	0.80	0.0497 (0.0021, 0.0026)	2.12 (2.35, 0.66)	4.61 (4.00, 0.78)
Desulfotalea psychrophila LSv54	265	0.85	0.0358 (0.0035, 0.0028)	2.62 (4.17, 1.03)	3.47 (4.33, 0.66)
Deinococcus radiodurans R1	258	0.78	0.0257 (0.0027, 0.0024)	2.19 (2.69, 0.69)	3.34 (3.74, 0.62)
Dictyostelium discoideum 2	252	0.75	0.0483 (0.0017, 0.0024)	1.28 (2.07, 0.57)	2.85 (3.78, 0.74)
Desulfovibrio vulgaris ssp. *vulgaris* Hildenborough	243	0.88	0.0360 (0.0042, 0.0030)	6.28 (5.34, 1.26)	5.28 (4.47, 0.62)
Escherichia coli K12	343	0.86	0.0389 (0.0029, 0.0023)	4.26 (4.58, 1.20)	5.53 (5.15, 0.80)
Enterococcus faecalis V583	229	0.80	0.0358 (0.0035, 0.0031)	2.89 (3.23, 0.95)	4.02 (3.89, 0.71)
Erwinia carotovora ssp. *atroseptica* SCRI1043	314	0.82	0.0469 (0.0029, 0.0025)	3.12 (3.92, 0.97)	4.48 (4.69, 0.73)
Encephalitozoon cuniculi	121	0.66	0.0337 (0.0016, 0.0029)	1.74 (1.57, 0.27)	2.51 (2.17, 0.35)
Fusarium graminearum 1	242	0.77	0.0472 (0.0022, 0.0026)	1.62 (2.88, 0.73)	3.06 (4.08, 0.69)
Fusobacterium nucleatum ssp. *nucleatum* ATCC 25586	215	0.77	0.0343 (0.0025, 0.0029)	2.07 (2.50, 0.60)	3.00 (3.38, 0.57)
Trypanosoma brucei	166	0.71	0.0442 (0.0022, 0.0031)	1.60 (2.26, 0.51)	2.51 (3.02, 0.51)
Leishmania major	173	0.68	0.0467 (0.0010, 0.0023)	0.88 (1.25, 0.25)	1.93 (2.52, 0.49)
3rd genome	191	0.73	0.0577 (0.0017, 0.0028)	1.19 (2.38, 0.63)	2.20 (3.45, 0.66)
Borrelia garinii PBi	118	0.73	0.0132 (0.0030, 0.0037)	2.39 (2.84, 0.58)	2.53 (2.68, 0.41)
Gallus gallus 22.1	403	1.09	0.0851 (0.0062, 0.0034)	10.48 (16.23, 1.54)	5.72 (5.98, 0.38)
Candida glabrata	187	0.67	0.0418 (0.0012, 0.0023)	0.92 (1.55, 0.35)	2.01 (2.96, 0.57)
Ashbya gossypii 1.0	198	0.68	0.0460 (0.0010, 0.0022)	0.81 (1.24, 0.28)	1.93 (2.70, 0.53)
Magnaporthe grisea 7 r2.3	220	0.72	0.0562 (0.0019, 0.0028)	0.94 (1.70, 0.44)	2.04 (3.13, 0.60)
Geobacter sulfurreducens PCA	291	0.86	0.0395 (0.0035, 0.0025)	4.42 (3.73, 1.00)	5.70 (4.48, 0.77)
Gloeobacter violaceus PCC 7421	287	0.83	0.0405 (0.0024, 0.0022)	3.78 (4.33, 1.22)	5.74 (5.01, 0.87)
Halobacterium sp. NRC-1	190	0.78	0.0334 (0.0026, 0.0029)	1.61 (3.20, 0.87)	2.50 (3.81, 0.69)

Haemophilus ducreyi 35000HP	212	0.75	0.0326 (0.0024, 0.0027)	2.76 (2.34, 0.55)	4.04 (3.29, 0.54)
Helicobacter hepaticus ATCC 51449	213	0.71	0.0298 (0.0013, 0.0021)	1.31 (1.56, 0.37)	2.72 (2.98, 0.56)
Haemophilus influenzae Rd KW20	238	0.74	0.0295 (0.0020, 0.0023)	2.35 (2.04, 0.52)	4.03 (3.38, 0.59)
Bartonella henselae Houston-1	186	0.76	0.0322 (0.0021, 0.0029)	2.51 (2.34, 0.59)	3.72 (3.24, 0.60)
Helicobacter pylori 26695	184	0.68	0.0349 (0.0015, 0.0024)	1.62 (1.80, 0.38)	2.77 (2.86, 0.46)
Homo sapiens 22.34d	389	0.99	0.0639 (0.0038, 0.0028)	10.62 (12.17, 1.62)	7.30 (6.59, 0.56)
Kluyveromyces lactis	194	0.65	0.0311 (0.0011, 0.0020)	0.85 (1.28, 0.26)	2.04 (2.67, 0.46)
Bacillus thuringiensis ser. *konkukian* 97-27	313	0.88	0.0388 (0.0042, 0.0027)	4.75 (5.13, 1.23)	4.79 (4.80, 0.69)
Kluyveromyces waltii	192	0.64	0.0388 (0.0009, 0.0021)	0.77 (1.05, 0.20)	1.95 (2.42, 0.43)
Lactobacillus plantarum WCFS1	216	0.79	0.0333 (0.0031, 0.0030)	2.67 (2.84, 0.77)	3.38 (3.56, 0.62)
Listeria innocua Clip11262	268	0.81	0.0335 (0.0037, 0.0028)	3.31 (3.78, 0.98)	4.24 (4.11, 0.64)
Lactobacillus johnsonii NCC 533	181	0.76	0.0233 (0.0022, 0.0027)	1.94 (2.71, 0.67)	2.75 (3.41, 0.61)
Lactococcus lactis ssp. *lactis* Il1403	218	0.80	0.0436 (0.0024, 0.0028)	2.97 (3.05, 0.88)	4.16 (3.95, 0.73)
Listeria monocytogenes EGD-e	259	0.81	0.0361 (0.0041, 0.0032)	3.44 (3.80, 1.00)	4.09 (4.11, 0.68)
Leptospira interrogans ser. Lai 56601	221	0.80	0.0443 (0.0026, 0.0027)	1.77 (2.18, 0.52)	3.47 (3.34, 0.59)
Leifsonia xyli ssp. *xyli* CTCB07	194	0.72	0.0238 (0.0015, 0.0022)	1.20 (1.97, 0.52)	2.24 (3.31, 0.66)
Mycoplasma mobile 163K	93	0.73	0.0151 (0.0028, 0.0040)	2.28 (3.44, 0.70)	2.00 (2.60, 0.39)
Methanococcus maripaludis S2	186	0.76	0.0236 (0.0024, 0.0028)	1.52 (2.80, 0.72)	2.37 (3.47, 0.62)
Methanosarcina acetivorans C2A	227	0.80	0.0490 (0.0026, 0.0028)	1.46 (2.75, 0.70)	2.57 (3.73, 0.64)
Mycobacterium tuberculosis H37Rv	273	0.85	0.0440 (0.0031, 0.0026)	3.60 (4.27, 1.19)	4.49 (4.64, 0.79)
Mycobacterium bovis AF2122/97	271	0.85	0.0448 (0.0029, 0.0026)	3.65 (4.16, 1.21)	4.51 (4.65, 0.83)
Methanothermobacter thermautotrophicus Delta H	190	0.79	0.0345 (0.0038, 0.0033)	3.18 (4.62, 1.10)	3.69 (4.20, 0.64)
Mycoplasma penetrans HF-2	108	0.69	0.0192 (0.0014, 0.0028)	1.69 (2.35, 0.47)	2.02 (2.50, 0.42)
Mesoplasma florum L1	107	0.76	0.0228 (0.0026, 0.0038)	2.10 (2.99, 0.64)	2.21 (2.72, 0.45)
Mycoplasma genitalium G-37	90	0.73	0.0156 (0.0031, 0.0045)	2.38 (3.34, 0.64)	1.99 (2.45, 0.36)
Mycoplasma mycoides ssp. *mycoides* SC PG1	110	0.73	0.0162 (0.0018, 0.0029)	1.98 (2.75, 0.64)	2.24 (2.76, 0.51)
Methanocaldococcus jannaschii DSM 2661	161	0.77	0.0298 (0.0024, 0.0029)	1.86 (3.40, 0.87)	2.41 (3.61, 0.63)
Mesorhizobium loti MAFF303099	298	0.88	0.0441 (0.0039, 0.0028)	4.34 (5.81, 1.45)	4.57 (5.19, 0.75)
Mycobacterium leprae TN	209	0.74	0.0388 (0.0017, 0.0024)	1.25 (1.81, 0.45)	2.36 (3.15, 0.58)
Mus musculus 22.32b	416	1.02	0.0807 (0.0036, 0.0030)	10.66 (11.97, 1.77)	7.80 (6.88, 0.65)
Methanopyrus kandleri AV19	147	0.73	0.0260 (0.0019, 0.0025)	1.16 (1.89, 0.40)	1.76 (2.62, 0.45)
Mycoplasma pneumoniae M129	92	0.73	0.0154 (0.0025, 0.0036)	2.19 (3.44, 0.67)	1.93 (2.58, 0.39)
Mycoplasma pulmonis UAB CTIP	102	0.72	0.0129 (0.0017, 0.0027)	1.93 (2.66, 0.52)	2.00 (2.46, 0.40)
Mycoplasma gallisepticum R	86	0.70	0.0164 (0.0020, 0.0038)	1.94 (2.87, 0.56)	1.79 (2.41, 0.38)
Methanosarcina mazei Go1	210	0.83	0.0462 (0.0030, 0.0031)	1.77 (3.34, 0.88)	2.84 (3.91, 0.69)
Nanoarchaeum equitans Kin4-M	57	0.68	0.0200 (0.0024, 0.0052)	2.57 (3.15, 0.64)	1.87 (2.17, 0.43)
Nitrosomonas europaea ATCC 19718	256	0.78	0.0392 (0.0026, 0.0026)	2.05 (2.65, 0.64)	3.67 (3.71, 0.60)
Neisseria meningitidis MC58	221	0.77	0.0408 (0.0022, 0.0025)	2.28 (2.18, 0.50)	3.76 (3.26, 0.53)
Nostoc sp. PCC 7120	289	0.88	0.0648 (0.0025, 0.0028)	2.56 (4.48, 1.29)	4.82 (5.19, 0.92)

Neurospora crassa 3	218	0.72	0.0393 (0.0014, 0.0024)	1.06 (1.35, 0.29)	2.38 (2.79, 0.50)
Oceanobacillus iheyensis HTE831	279	0.82	0.0274 (0.0035, 0.0026)	4.87 (3.72, 0.96)	4.99 (4.29, 0.68)
Oryza sativa ssp. *japonica* 2.0	343	0.98	0.0782 (0.0061, 0.0035)	8.39 (11.16, 1.44)	5.62 (5.54, 0.46)
Onion yellows phytoplasma OY-M	95	0.76	0.0115 (0.0028, 0.0038)	2.42 (4.09, 0.96)	2.09 (2.96, 0.51)
Parachlamydia sp. UWE25	183	0.74	0.0237 (0.0022, 0.0027)	1.68 (2.43, 0.57)	2.63 (3.21, 0.56)
Picrophilus torridus DSM 9790	160	0.72	0.0288 (0.0020, 0.0027)	1.53 (2.33, 0.54)	2.37 (3.00, 0.49)
Pseudomonas aeruginosa PAO1	335	0.93	0.0552 (0.0041, 0.0029)	7.03 (6.53, 1.59)	6.13 (5.50, 0.80)
Pyrococcus abyssi GE5	177	0.77	0.0251 (0.0025, 0.0029)	1.70 (2.69, 0.71)	2.52 (3.35, 0.63)
Pyrobaculum aerophilum IM2	175	0.73	0.0175 (0.0026, 0.0029)	1.59 (2.54, 0.59)	2.42 (3.22, 0.54)
Photorhabdus luminescens ssp. *laumondii* TTO1	311	0.84	0.0459 (0.0028, 0.0025)	3.62 (3.40, 0.92)	4.87 (4.49, 0.75)
Porphyromonas gingivalis W83	203	0.76	0.0386 (0.0025, 0.0028)	2.12 (2.45, 0.63)	2.93 (3.31, 0.61)
Pyrococcus horikoshii OT3	167	0.76	0.0329 (0.0024, 0.0032)	1.53 (2.34, 0.56)	2.40 (3.14, 0.57)
Pirellula sp. 1	260	0.83	0.0423 (0.0031, 0.0027)	1.73 (3.74, 1.00)	2.90 (4.41, 0.74)
Plasmodium falciparum 1	110	0.61	0.0290 (0.0009, 0.0025)	1.19 (1.45, 0.24)	1.90 (2.20, 0.39)
Pasteurella multocida Pm70	250	0.76	0.0335 (0.0021, 0.0024)	2.63 (2.43, 0.66)	4.36 (3.73, 0.65)
Schizosaccharomyces pombe	225	0.72	0.0366 (0.0017, 0.0024)	1.36 (1.75, 0.43)	2.70 (3.15, 0.59)
Prochlorococcus marinus ssp. *marinus* CCMP1375	187	0.73	0.0273 (0.0019, 0.0025)	1.59 (2.36, 0.62)	2.63 (3.40, 0.62)
Pseudomonas putida KT2440	314	0.91	0.0467 (0.0042, 0.0028)	5.45 (6.38, 1.38)	4.85 (5.15, 0.70)
Pseudomonas syringae pv. *tomato* DC3000	318	0.90	0.0550 (0.0041, 0.0029)	4.68 (5.44, 1.39)	4.72 (4.96, 0.75)
Pyrococcus furiosus DSM 3638	177	0.79	0.0400 (0.0029, 0.0032)	1.71 (3.16, 0.85)	2.49 (3.59, 0.65)
Plasmodium yoelii ssp. *yoelii* 1	117	0.64	0.0178 (0.0011, 0.0028)	1.35 (1.48, 0.24)	2.04 (2.10, 0.33)
Phytophthora ramorum 1.0	317	0.80	0.0688 (0.0025, 0.0030)	1.47 (3.15, 0.88)	3.12 (4.72, 0.86)
Rickettsia conorii Malish 7	151	0.72	0.0193 (0.0025, 0.0032)	2.25 (2.39, 0.51)	2.82 (2.78, 0.43)
Rhodopseudomonas palustris CGA009	280	0.85	0.0483 (0.0031, 0.0027)	2.99 (4.21, 1.22)	4.11 (4.73, 0.82)
Rhodobacter sphaeroides	269	0.80	0.0355 (0.0022, 0.0024)	2.09 (2.26, 0.61)	3.63 (3.73, 0.68)
Rattus norvegicus 22.3b	428	1.04	0.0713 (0.0038, 0.0028)	9.74 (13.68, 1.54)	6.78 (6.62, 0.52)
Rickettsia prowazekii Madrid E	142	0.75	0.0305 (0.0030, 0.0036)	4.26 (3.12, 0.66)	4.27 (2.98, 0.45)
Ralstonia solanacearum GMI1000	274	0.80	0.0360 (0.0025, 0.0024)	2.77 (2.85, 0.75)	4.27 (4.11, 0.70)
Rickettsia typhi wilmington	142	0.73	0.0273 (0.0024, 0.0032)	2.44 (2.60, 0.50)	2.74 (2.74, 0.39)
Shigella flexneri 2a 301	326	0.84	0.0360 (0.0027, 0.0023)	4.43 (4.20, 1.07)	5.63 (4.96, 0.76)
Staphylococcus epidermidis ATCC 12228	241	0.78	0.0361 (0.0028, 0.0027)	2.90 (3.20, 0.79)	4.24 (3.91, 0.62)
Streptococcus agalactiae 2603V/R	219	0.80	0.0383 (0.0026, 0.0026)	2.14 (3.31, 0.85)	3.32 (3.90, 0.64)
Streptococcus mutans UA159	211	0.80	0.0395 (0.0024, 0.0026)	1.93 (3.06, 0.96)	3.24 (3.98, 0.83)
Streptomyces avermitilis MA-4680	311	0.95	0.0552 (0.0048, 0.0030)	5.67 (8.31, 1.76)	4.93 (5.66, 0.73)
Staphylococcus aureus ssp. *aureus* Mu50	257	0.81	0.0351 (0.0032, 0.0028)	3.16 (3.36, 0.82)	4.15 (3.99, 0.62)
Symbiobacterium thermophilum IAM 14863	266	0.84	0.0394 (0.0033, 0.0026)	4.61 (4.42, 1.15)	5.04 (4.63, 0.76)
Saccharomyces cerevisiae	193	0.72	0.0415 (0.0016, 0.0025)	1.09 (1.74, 0.40)	2.32 (3.09, 0.58)
Streptomyces coelicolor A3(2)	309	0.99	0.0587 (0.0051, 0.0031)	5.24 (8.87, 1.47)	4.46 (5.52, 0.60)
Salmonella enterica ssp. *enterica* ser. Typhi CT18	312	0.84	0.0444 (0.0032, 0.0026)	4.36 (4.66, 1.21)	5.04 (4.86, 0.75)

Phytophthora sojae 1.0	282	0.82	0.0705 (0.0027, 0.0031)	1.05 (3.23, 0.89)	2.28 (4.42, 0.77)
Shewanella oneidensis MR-1	312	0.84	0.0461 (0.0031, 0.0026)	3.59 (4.03, 1.11)	4.81 (4.72, 0.79)
Sinorhizobium meliloti 1021	271	0.82	0.0428 (0.0023, 0.0024)	2.61 (2.97, 0.83)	4.13 (4.26, 0.76)
Streptococcus pyogenes MGAS10394	201	0.81	0.0344 (0.0030, 0.0027)	3.24 (3.76, 1.05)	3.98 (4.01, 0.70)
Streptococcus pyogenes M1 GAS	197	0.79	0.0364 (0.0029, 0.0029)	3.42 (3.48, 0.98)	4.09 (3.88, 0.68)
Streptococcus pneumoniae TIGR4	214	0.76	0.0391 (0.0017, 0.0024)	1.63 (2.24, 0.64)	3.14 (3.55, 0.73)
Sulfolobus solfataricus P2	187	0.76	0.0256 (0.0020, 0.0025)	1.21 (2.14, 0.58)	2.12 (3.17, 0.62)
Salmonella typhimurium LT2	314	0.86	0.0453 (0.0032, 0.0026)	4.77 (4.92, 1.25)	5.33 (5.08, 0.79)
Sulfolobus tokodaii 7	187	0.78	0.0304 (0.0021, 0.0026)	1.43 (2.34, 0.67)	2.46 (3.33, 0.67)
Synechococcus sp. WH 8102	235	0.69	0.0233 (0.0011, 0.0017)	1.08 (1.34, 0.31)	2.68 (2.97, 0.55)
Thermus thermophilus HB27	253	0.78	0.0287 (0.0027, 0.0025)	2.29 (3.42, 0.85)	3.34 (4.12, 0.65)
Thermoplasma acidophilum DSM 1728	160	0.78	0.0389 (0.0029, 0.0033)	1.64 (3.49, 0.93)	2.32 (3.61, 0.66)
Trypanosoma brucei	15	0.53	0.1000 (0.0010, 0.0099)	5.71 (5.63, 0.55)	1.42 (1.40, 0.15)
Treponema denticola ATCC 35405	199	0.72	0.0273 (0.0023, 0.0026)	1.59 (2.13, 0.50)	2.56 (3.06, 0.48)
Clostridium tetani E88	241	0.83	0.0227 (0.0033, 0.0027)	2.86 (3.16, 0.79)	3.46 (3.74, 0.60)
Thermosynechococcus elongatus BP-1	251	0.81	0.0440 (0.0024, 0.0026)	2.15 (3.26, 0.83)	3.91 (4.26, 0.76)
Thalassiosira pseudonana	322	0.76	0.0507 (0.0022, 0.0024)	1.57 (2.63, 0.66)	3.21 (4.18, 0.68)
Thermotoga maritima MSB8	225	0.84	0.0386 (0.0032, 0.0029)	2.47 (4.42, 1.19)	3.13 (4.42, 0.75)
Fugu rubripes 22.2c	435	1.06	0.0697 (0.0048, 0.0029)	10.71 (16.22, 1.41)	5.90 (6.22, 0.36)
Treponema pallidum ssp. *pallidum* Nichols	139	0.69	0.0220 (0.0015, 0.0025)	1.69 (1.98, 0.41)	2.44 (2.58, 0.43)
Thermoanaerobacter tengcongensis	264	0.84	0.0361 (0.0042, 0.0030)	4.19 (4.42, 1.17)	4.50 (4.37, 0.70)
Thermoplasma volcanium GSS1	155	0.80	0.0287 (0.0039, 0.0036)	1.91 (4.28, 1.10)	2.38 (3.72, 0.62)
Tropheryma whipplei Twist	147	0.69	0.0409 (0.0011, 0.0024)	1.26 (1.47, 0.33)	2.24 (2.47, 0.50)
Ustilago maydis 1 r2	150	0.71	0.0493 (0.0019, 0.0031)	1.30 (2.03, 0.47)	2.05 (2.90, 0.54)
Ureaplasma parvum ser. 3 ATCC 700970	82	0.72	0.0131 (0.0031, 0.0046)	2.45 (3.35, 0.66)	2.04 (2.43, 0.36)
Vibrio vulnificus YJ016	323	0.87	0.0382 (0.0030, 0.0024)	4.82 (4.97, 1.27)	4.99 (5.25, 0.82)
Vibrio cholerae O1 *biovar eltor* N16961	303	0.81	0.0477 (0.0027, 0.0025)	4.29 (4.33, 1.07)	4.82 (4.82, 0.73)
Chromobacterium violaceum ATCC 12472	308	0.87	0.0516 (0.0028, 0.0026)	3.22 (4.30, 1.19)	4.53 (4.96, 0.85)
Vibrio parahaemolyticus RIMD 2210633	318	0.87	0.0435 (0.0035, 0.0027)	5.12 (5.60, 1.33)	4.95 (5.12, 0.74)
Vibrio vulnificus CMCP6	325	0.86	0.0419 (0.0032, 0.0025)	3.98 (5.26, 1.24)	4.76 (5.22, 0.77)
Wigglesworthia glossinidia	161	0.69	0.0332 (0.0016, 0.0025)	2.44 (2.00, 0.44)	3.69 (2.77, 0.45)
Wolbachia	167	0.69	0.0206 (0.0015, 0.0024)	1.61 (1.80, 0.36)	2.57 (2.69, 0.44)
Wolinella succinogenes DSM 1740	233	0.76	0.0425 (0.0022, 0.0026)	1.49 (2.60, 0.66)	3.27 (3.76, 0.63)
Xanthomonas axonopodis pv. *citri* 306	299	0.89	0.0466 (0.0031, 0.0025)	3.38 (4.90, 1.28)	4.45 (4.96, 0.76)
Xanthomonas campestris pv. *campestris* ATCC 33913	303	0.88	0.0421 (0.0033, 0.0026)	4.92 (5.65, 1.40)	5.03 (5.18, 0.75)
Xenopus tropicalis 2.0	548	1.39	0.0895 (0.0113, 0.0035)	27.26 (30.73, 1.33)	5.49 (5.33, 0.12)
Xylella fastidiosa 9a5c	249	0.80	0.0366 (0.0025, 0.0027)	2.33 (2.52, 0.64)	3.81 (3.67, 0.64)
Pan troglodytes 22.1	406	1.07	0.0703 (0.0053, 0.0031)	13.04 (16.24, 1.68)	6.89 (6.32, 0.42)
Saccharomyces bayanus MIT	209	0.67	0.0471 (0.0010, 0.0022)	0.82 (1.46, 0.35)	1.97 (3.05, 0.61)

Saccharomyces mikatae MIT	200	0.68	0.0355 (0.0013, 0.0023)	0.94 (1.41, 0.32)	2.12 (2.79, 0.53)
Saccharomyces paradoxus MIT	197	0.68	0.0309 (0.0015, 0.0023)	0.95 (1.56, 0.35)	2.05 (2.90, 0.52)
Yarrowia lipolytica	208	0.72	0.0431 (0.0014, 0.0024)	1.11 (1.71, 0.40)	2.33 (3.18, 0.62)
Yersinia pestis CO92	302	0.85	0.0547 (0.0032, 0.0027)	3.38 (4.46, 1.13)	4.51 (4.80, 0.75)
Yersinia pseudotuberculosis IP 32953	309	0.86	0.0490 (0.0033, 0.0027)	3.34 (4.46, 1.19)	4.48 (4.87, 0.79)
